# Studies on irritable bowel syndrome associated with anxiety or depression in the last 20 years: A bibliometric analysis

**DOI:** 10.3389/fpubh.2022.947097

**Published:** 2022-08-15

**Authors:** Yuanfang Chen, Baotao Lian, Peize Li, Simeng Yao, Zhengkun Hou

**Affiliations:** ^1^The First Clinical Medical College, Guangzhou University of Chinese Medicine, Guangzhou, China; ^2^Department of Intensive Care Unit, The Second Affiliated Hospital of Guangzhou University of Chinese Medicine (Guangdong Provincial Hospital of Chinese Medicine), Guangzhou, China; ^3^Department of Gastroenterology, The First Affiliated Hospital of Guangzhou University of Chinese Medicine, Guangzhou, China

**Keywords:** irritable bowel syndrome, anxiety, depression, bibliometrics, citespace

## Abstract

Irritable bowel syndrome (IBS) associated with anxiety or depression is ubiquitous in clinical practice, and multiple related articles have been published. However, studies that utilize bibliometric analyses to address this topic are rare. In our study, we aimed to reveal research trends in IBS with anxiety or depression. Publications on IBS in relation to anxiety or depression in the last 20 years were obtained from the Web of Science Core Collection (WoSCC). CiteSpace software (5.8.R3) and GraphPad Prism 8 were used to perform bibliometric analysis of authors, countries, institutions, journals, keywords, and references involved in this topic. A total of 2,562 publications from 716 academic journals were included in this study. The majority of publications (*n* = 833, 32.51%) were from the USA, and the University of California, Los Angeles, contributed the most publications (*n* = 97, 3.79%). Active cooperations among countries and institutions were observed. *Neurogastroenterology and Motility* [impact factor (IF) 2020 = 3.598] published the most papers (170 publications, 6.64%), followed by *Alimentary Pharmacology Therapeutics* (IF 2020 = 8.171; 88 publications; 3.44%). The literatures related to IBS and anxiety or depression were primarily published in journals related to medicine/medical/clinical, neurology/sports/ophthalmology, and molecular/biology/immunology. Cryan JF and Drossman DA, with the largest number of articles (84 publications) and citations (917 citations), respectively, were considered as the most influential authors in this field. A total of 336 co-cited references were divided into 17 clusters, and #1 *fecal microbiota transplantation* contained most of the documents published in recent years. Moreover, the keyword “psychosocial factor” had the largest burst strength of 13.52, followed by the keyword “gut microbiota” with a burst strength of 11.71. This study shows the research performance of IBS related to anxiety or depression from 2002 to 2021 and helps researchers master the trend in this field, which should receive more attention.

## Introduction

Irritable bowel syndrome (IBS) is a functional gastrointestinal disorder which is typified by recurrent abdominal pain and changes in bowel habit, as described in the Rome diagnostic criteria (1). Data show that IBS affects ~11% of the population globally (2); however, variations exist in the prevalence of IBS among countries and regions. As a relapsing chronic disease in which symptoms may change over time, IBS seriously affects the quality of life (3). Patients with IBS agonize over the unpredictability of their symptoms and feel a loss of freedom and spontaneity in their work and life (4–6). In addition, IBS entails a significant financial burden for individual patients, society, and the healthcare system (4). According to an analysis based on 35 studies, the direct costs of IBS in the USA, including outpatient costs, inpatient costs, and drug costs, are between $1,562 and $7,547 per patient per year (7). It is also reported that in the USA, absenteeism caused by illness costs employers an average of $901 each year per employee with IBS (8). Unfortunately, the treatment shows limited effect as the cause of IBS has not been completely elucidated. So far, IBS remains a clinical challenge with an increase in the number of patients (9).

Anxiety disorder and depression are the most prevalent classes of psychiatric conditions, and they are included in the top 10 largest contributors to global disability (10). Based on a report by the World Health Organization, the global prevalence of depression was estimated to be 4.4% and the prevalence of anxiety was 3.6%, indicating that 322 million of people in the world are suffering from depression and 264 million of the population are affected by anxiety disorder. Previous research has evidenced that IBS is closely related to anxiety and depression. Firstly, they have been proved to have a high probability to be accompanied. Studies manifested that 39.1% of IBS patients had anxiety symptoms and 28.8% of them had depressive symptoms (11). Compared to healthy individuals, IBS patients were three times more likely to suffer from anxiety or depression (11). A recent systematic review with meta-analysis also pointed out that the prevalence of anxiety and depression is the highest in individuals with IBS with constipation (38 and 40%, respectively) among all the subtypes of IBS (12). Besides, numerous studies have confirmed that anxiety and depression are closely related to the initiation, development, and progression of IBS (13, 14). In the meantime, the two mental disorders might also exacerbate both gastrointestinal and extra-gastrointestinal symptoms by altering visceral sensation, changing the gut microenvironment, and influencing the microbiome–gut–brain axis mainly, which further impair quality of life and add complexity to the management of IBS patients (15–17). Psychological interventions such as cognitive behavioral therapy, hypnotherapy, multicomponent psychological therapy, relaxation therapy, and dynamic psychotherapy are confirmed to be effective in the treatment of IBS (18). With the development of gastroenterology and psychosomatic medicine, increasing attention has been paid to the relationship between IBS and anxiety/depression. Understanding the research trends and hotspots in this field at present time is helpful for us to carry out in-depth research which may provide new insight into the therapy of patients with IBS.

Presently, bibliometric analysis, as a tool combining data mining and visualization, contributes to evaluating the scientific research quantitatively and qualitatively and predicting the research trends in specific fields (19). To delve into the global trend of researches related to IBS and anxiety/depression, this study used CiteSpace (20), a bibliometric analysis software, to construct visualization maps in order to intuitively display valuable information hiding under detailed information of indexes such as authors, institutions, countries, journals, keywords, or any other data. Nevertheless, there are few bibliometric studies on IBS and anxiety/depression. This study used the bibliometric method to conduct a comprehensive analysis of global publications about IBS associated with anxiety or depression in the recent 20 years *via* CiteSpace, aiming to shed new light for research on the discovery of new treatments and management of IBS.

## Materials and methods

### Data collection and search strategy

A comprehensive search was conducted using the Science Citation Index Expanded (SCI-E) in the Web of Science Core Collection (WoSCC) database on 14 February 2022 at Guangzhou University of Chinese Medicine, Guangdong, China. The following search terms were employed: topics = (“anxiety” OR “depression” OR “depressive disorder” OR “mental health disorder*” OR “psychiatric disorder*”) AND topics = (IBS OR “irritable bowel syndrome^*^” OR “irritable bowel” OR “irritable colon”). Publications between 2002 and 2021 were identified, and the language of the publications was restricted to English. In this study, documents defined as “article” or “review” were included only. Data from WoSCC were initially downloaded in txt format and verified by two independent reviewers (Baotao Lian and Peize Li). Any differences between the two reviewers were settled through discussion with a third reviewer (Simeng Yao).

### Analysis tool

Statistical analysis was performed using GraphPad Prism 8, and bibliometric analysis was carried out by version 5.8.R3 of CiteSpace software. Developed by Chen Chaomei, CiteSpace is a Java-based bibliometric analysis visualization software which is completely free to use (21, 22). It was used in this study to analyze the cooperation network of countries, institutions, keywords, co-cited references, and authorship so that the structure of a field or fields could be revealed (23). Nodes in the network map performed by CiteSpace represented countries, institutions, authors, keywords, or co-cited references. The size of a node indicated the frequency of occurrence or citation, and the color of it represented different clusters or years. Network edges in the map stand for cooperation, co-occurrence, and co-citation relationships, and the color of a network edge indicated the year in which the cooperation, co-occurrence, or co-citation link was first set up. Dual-map overlay for journals, cluster detection, and citation bursts detection for references were also carried out by CiteSpace.

In addition, GraphPad Prism was used to analyze the trend of publication. A polynomial model (*Y* = B0 + B1^*^(*X* – Mean*X*) + B2^*^[(*X* – Mean*X*)^2^) + B3^*^[(*X* – Mean*X*)^3^)] was conducted in order to predict the number of published papers in research related to IBS and anxiety or depression in 2022. In this case, variable *X* stands for the publication year and *Y* stands for the number of publications.

## Results

### Annual publications and growth forecast

**Figure 1:**
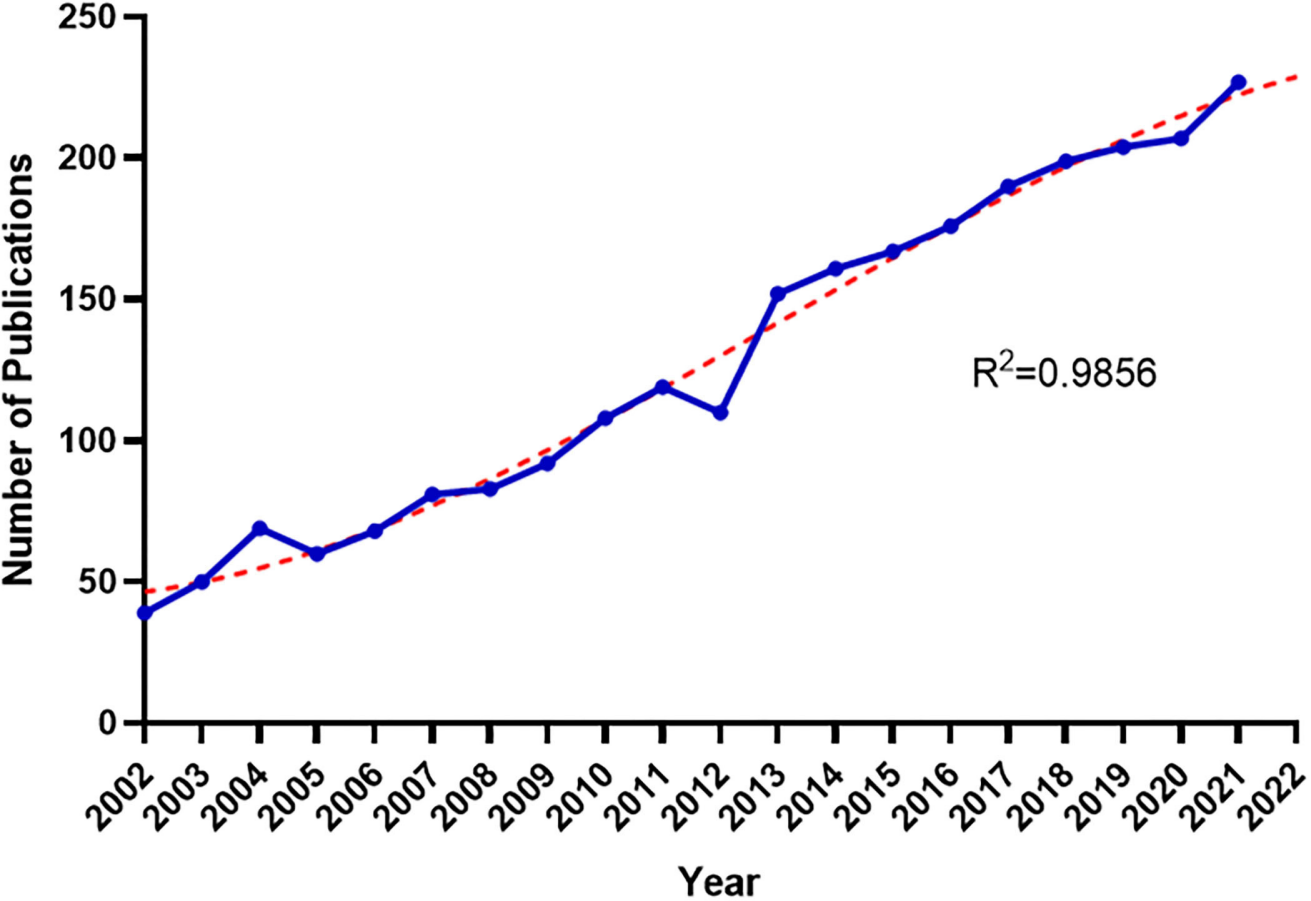
Annual publications and growth forecast of IBS research related to anxiety or depression.

The search queries are listed in Table 1. The search strategy recovered 2,562 papers, including 2,053 articles and 509 reviews. Figure 1 shows the number of papers published per year since 2002, indicating that there has been a steady rise in the number of publications. In 2002, only 39 works related to IBS and anxiety or depression were published; however, two decades later in 2021, the annual publications were almost six times the previous amount. The yearly number of published articles reached a peak in 2021 (*n* = 227). The remarkable growth in the number of publications in the recent 20 years implied increasing attention on IBS associated with anxiety or depression.

**Table 1:** Data sources.

**Content**			
Data sources	WoSCC(SCI-E)		
Languages	English only		
Year of publication	2002–2021		
Document type	Article or review		
Search strategy	#1	387,859	TS=(anxiety) OR TS=(depression) OR TS=(“depressive disorder”) OR TS=(“mental health disorder*”) OR TS=(“psychiatric disorder*”)
	#2	16,552	TS=(IBS) OR TS=(“irritable bowel syndrome*”) OR TS=(“irritable bowel”) OR TS=(“irritable colon”)
	#3	2,562	#1 & #2

In addition, a growth trend model was performed [the coefficient of determination (*R*^2^) = 0.9856], and it showed a significant correlation between publication year and the number of publications (Figure 1). This model also predicted that the amount of literature related would reach 226 in 2022.

### Distribution of countries and institutions

**Figure 2:**
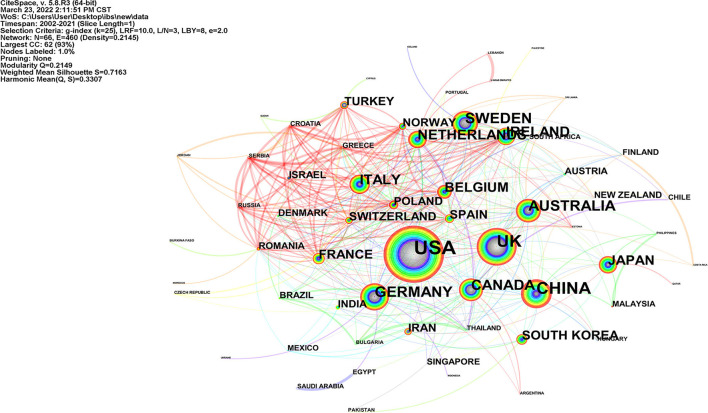
Collaboration network of countries in IBS research related to anxiety or depression.

Publications from England, Northern Ireland, Scotland, and Wales were reclassified to the UK, and those from Taiwan, Hong Kong, and Macao were assigned to China. Sixty-six countries contributed to 2,562 publications in the field of IBS and anxiety/depression research. The largest number of publications came from the USA (833, 32.51%), followed by China (316, 12.33%), UK (302, 11.79%), Sweden (172, 6.71%), and Australia (162, 6.32%; Table 2). As presented in Figure 2, the collaborations between countries are shown with 66 nodes and 460 links in a network map. The size of the nodes in the map represents the country's paper output, and the lines in the network represent the cooperative relationship between countries. There were active collaborations between the countries.

**Table 2:** Top 10 countries and institutions contributed to the publications.

**Rank**	**Count (%)**	**Country**	**Rank**	**Count (%)**	**Institution**
1	833 (32.51%)	USA	1	97 (3.79%)	University of California, Los Angeles
2	316 (12.33%)	China	2	96 (3.75%)	University College Cork
3	302 (11.79%)	UK	3	75 (2.93%)	University of North Carolina
4	172 (6.71%)	Sweden	4	62 (2.42%)	University of Washington
5	162 (6.32%)	Australia	5	56 (2.19%)	University of Gothenburg
6	156 (6.09%)	Germany	6	54 (2.11%)	Mayo clinic
7	143(5.58%)	Canada	7	51 (1.99%)	Karolinska Institutet
8	112 (4.37%)	Italy	8	41 (1.60%)	Newcastle University
9	112 (4.37%)	Netherlands	9	39 (1.52%)	McMaster University
10	108 (4.22%)	Ireland	10	35 (1.37%)	King's College London

**Figure 3:**
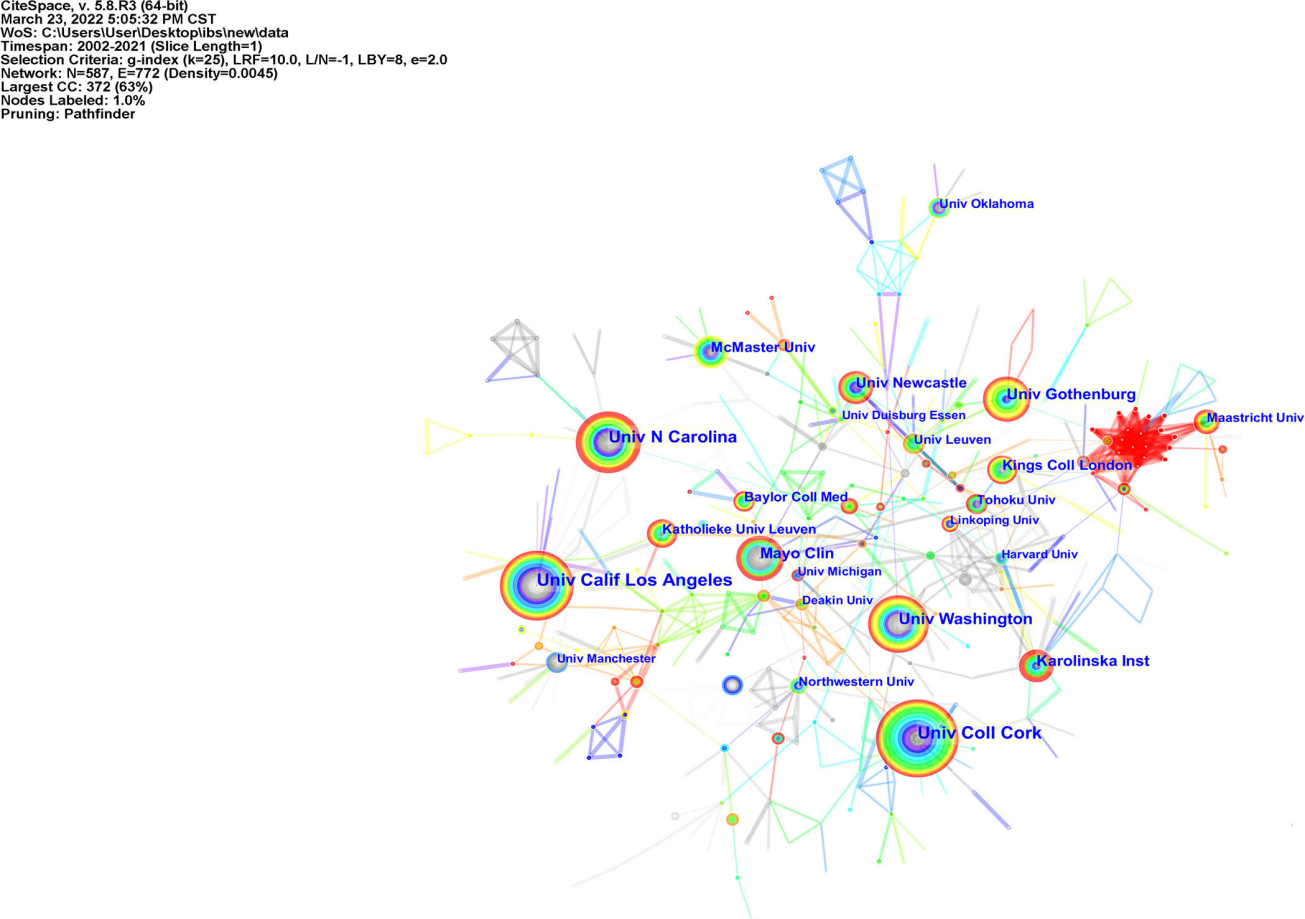
Collaboration network of institutions in IBS research related to anxiety or depression.

The publications on IBS and anxiety or depression were from 587 institutions. The top 10 institutions published 606 articles, about 23.65% of the total (Table 2). The University of California, Los Angeles (97, 3.79%), published the greatest number, followed by the University College Cork (96, 3.75%), University of North Carolina (75, 2.93%), and the University of Washington (62, 2.42%), University of Gothenburg (56, 2.19%). Apart from that, among the top 10 institutions with the most publications, four of them were from the USA, suggesting that the USA places considerable value on the IBS and anxiety/depression research. Figure 3 shows the networks between institutions and indicates that cooperation between institutions was active.

### Analysis of journals

In total, 716 academic journals have published papers in research related to IBS and anxiety or depression. Table 3 presents the top 10 most productive journals. *Neurogastroenterology and Motility* [impact factor (IF) 2020 = 3.598] published the most papers (170 publications, 6.64%), followed by *Alimentary Pharmacology Therapeutics* (IF 2020 =8.171; 88 publications; 3.44%), *American Journal of Gastroenterology* (IF 2020 = 10.864; 73 publications; 2.85%), *Journal of Psychosomatic Research* (IF 2020 = 3.006; 58 publications; 2.26%), and *World Journal of Gastroenterology* (IF 2020 = 5.742; 54 publications; 2.11%). Furthermore, among the top 10 journals, *Gut* (IF 2020 = 23.059), *Clinical Gastroenterology and Hepatology* (IF 2020 = 11.382), and *American Journal of Gastroenterology* (IF 2020 =10.864) had an IF higher than 10. Two of them (*Alimentary Pharmacology Therapeutics* and *World Journal of Gastroenterology*) had an IF between five and ten, and the rest of the top 10 journals had an IF between three and five.

**Table 3:** Top 10 journals contributed to the publications.

**Rank**	**Journal**	**Count (%)**	**IF 2020**
1	Neurogastroenterology and Motility	170 (6.64%)	3.598
2	Alimentary Pharmacology Therapeutics	88 (3.44%)	8.171
3	American Journal of Gastroenterology	73 (2.85%)	10.864
4	Journal of Psychosomatic Research	58 (2.26%)	3.006
5	World Journal of Gastroenterology	54 (2.11%)	5.742
6	PLOS ONE	51 (1.99%)	3.24
7	Clinical Gastroenterology and Hepatology	49 (1.91%)	11.382
8	Digestive Diseases and Sciences	48 (1.87%)	3.199
9	Gut	44 (1.72%)	23.059
10	Journal of Neurogastroenterology and Motility	43 (1.68%)	4.924

**Figure 4:**
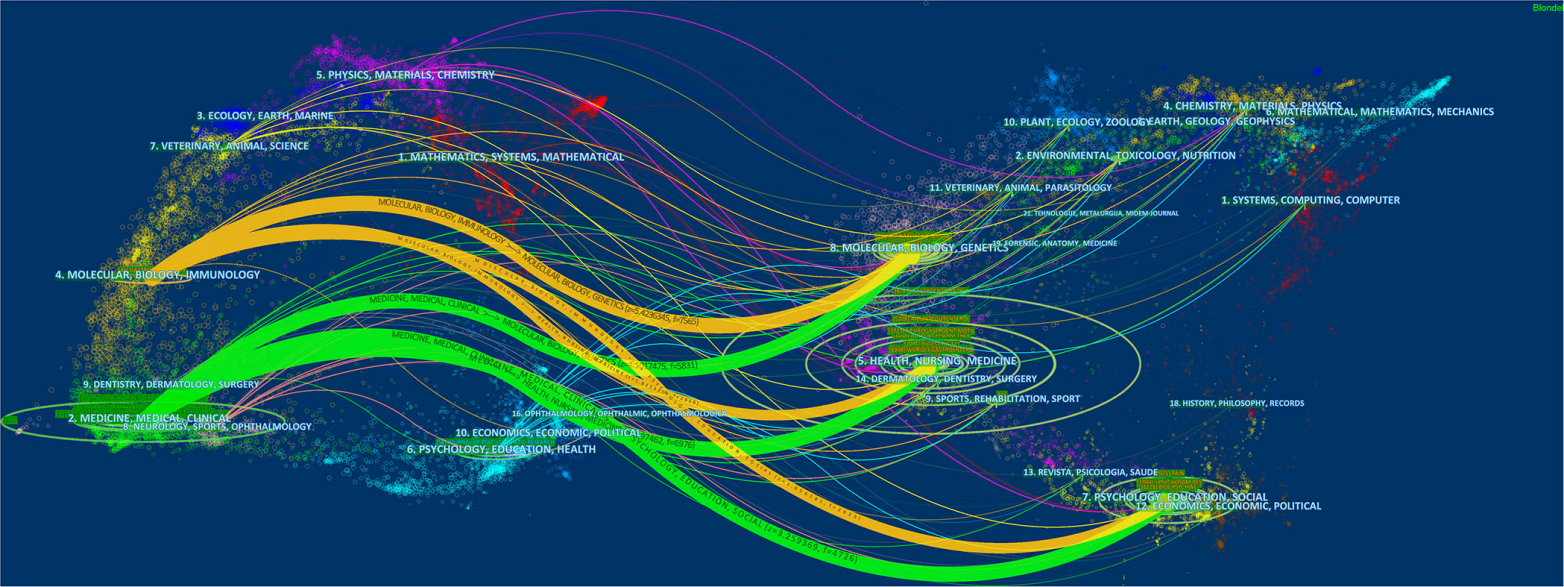
The dual-map overlay of journals.

Dual-map visualization was used in this study to provide a global visualization of the literature growth at a disciplinary level. On the left side of the map is the network of citing journals and the labels denote the disciplines of the citing journals. The network of cited journals is on the right-hand side of the dual map, and the labels on it represent the disciplines to which the cited references belong. The colored lines which begin from the citation map on the left and point to the cited map on the right represent the paths of the references. As presented in Figure 4, the literatures related to IBS and anxiety or depression are primarily published in journals related to medicine/medical/clinical (the area in green), neurology/sports/ophthalmology (the area in pink), and molecular/biology/immunology (the area in yellow). Meanwhile, the majority of publications were cited in journals related to molecular/biology/genetics, health/nursing/medicine, and psychology/education/social science.

### Analysis of authors

**Figure 5:**
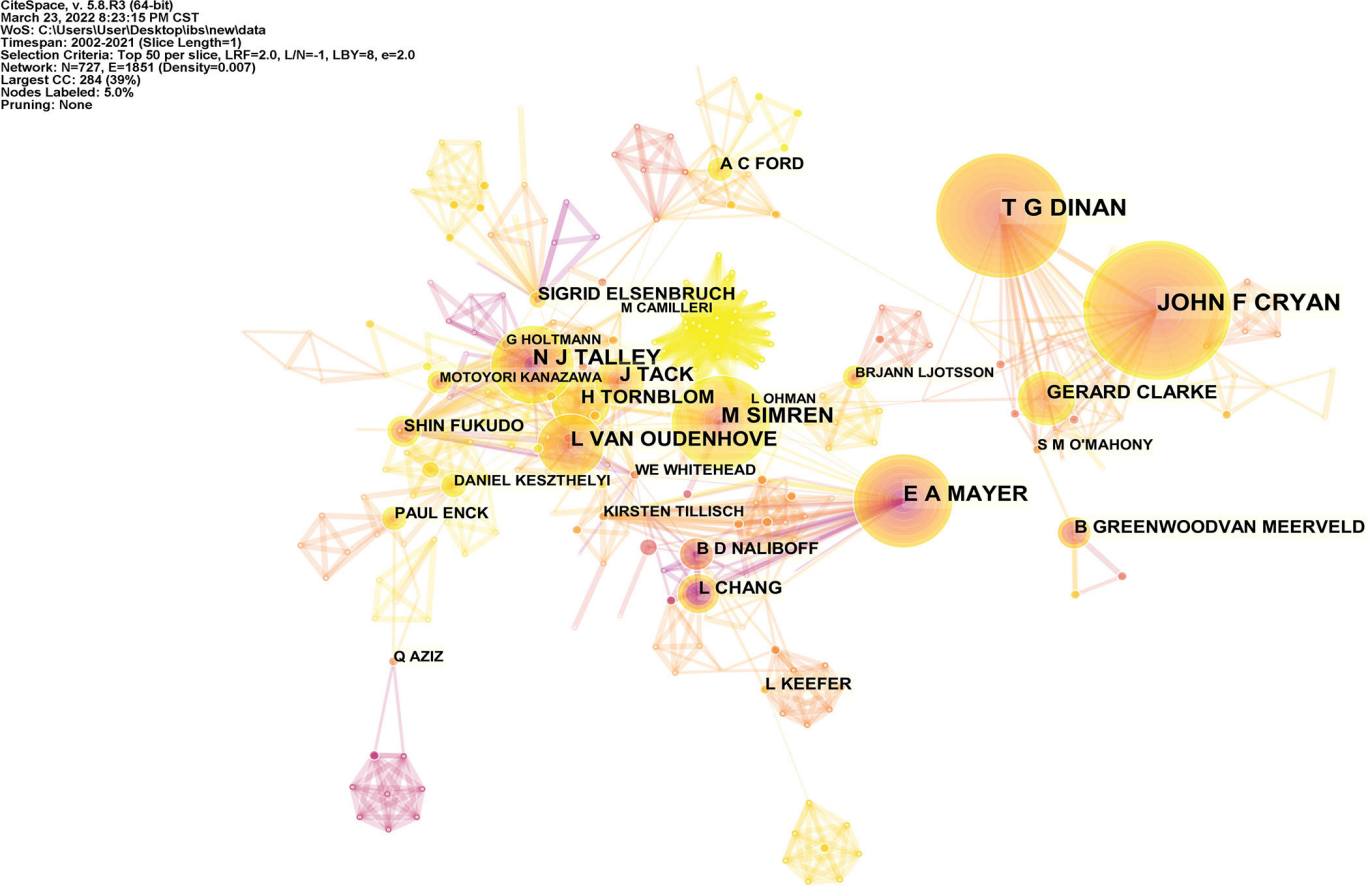
Authors' collaboration network of IBS research related to anxiety or depression.

There were over 10,000 authors who contributed to these 2,562 publications. Table 4 shows the top 10 authors and co-cited authors. Cryan JF made contributions to the largest number of articles (84 publications), followed by Dinan TG (81 publications) and Simrén M (63 publications). The top five cited authors were Drossman DA (917 citations), Whitehead WE (526 citations), Mayer EA (521 citations), Talley NJ (484 citations), and Longstreth GF (469 citations). Setting the threshold to the top 50 most productive authors in a 1-year slice, CiteSpace was used to produce the network of authors (Figure 5). The network map contained 727 nodes and 1,851 links, suggesting that there were active collaborations among the productive authors.

**Table 4:** Top 10 authors and co-cited authors of IBS and anxiety/depression research.

**Rank**	**Author**	**Count**	**Rank**	**Co-cited author**	**Citation**
1	Cryan JF	84	1	Drossman DA	917
2	Dinan TG	81	2	Whitehead WE	526
3	Simren M	63	3	Mayer EA	521
4	Talley NJ	54	4	Talley NJ	484
5	Mayer EA	53	5	Longstreth GF	469
6	Van Oudenhove L	47	6	Camilleri M	382
7	Tack J	37	7	Zigmond AS	372
8	Clarke G	36	8	Ford AC	320
9	Tornblom H	34	9	Thompson WG	273
10	Naliboff BD	31	10	Chang L	271

### Analysis of co-cited references

CiteSpace software visualizes the literature in the form of a co-citation network that reveals the structure of one or more fields through an analysis of article citations. Table 5 presents the top 10 co-cited references related to IBS with anxiety or depression. The number of citations for the top 10 co-cited references in this study varied from 150 to 83. The most co-cited reference was “Functional Bowel Disorders” published in Gastroenterology in 2006 (24), where Longstreth and his working team updated Rome II diagnostic criteria and treatment recommendations for the IBS and suggested that psychological disturbance should be identified as psychological factor of IBS and psychotherapy may be beneficial.

**Table 5:** Top 10 co-cited references on IBS and anxiety/depression.

**Rank**	**Citation**	**Author**	**Year**	**Title**	**Source**
1	150	Longstreth GF	2006	Functional bowel disorders	Gastroenterology
2	139	Lovell RM	2012	Global prevalence of and risk factors for irritable bowel syndrome: a meta-analysis	Clinical Gastroenterology and Hepatology
3	126	Lacy BE	2016	Bowel disorders	Gastroenterology
4	113	Fond G	2014	Anxiety and depression comorbidities in irritable bowel syndrome (IBS): a systematic review and meta-analysis	European Archives of Psychiatry and Clinical Neuroscience
5	112	Bravo JA	2011	Ingestion of lactobacillus strain regulates emotional behavior and central GABA receptor expression in a mouse *via* the Vagus nerve	Proceedings of the National Academy of Sciences of the United States of America
6	104	Drossman DA	2016	Functional gastrointestinal disorders: history, pathophysiology, clinical features, and Rome IV	Gastroenterology
7	99	O'Mahony SM	2009	Early life stress alters behavior, immunity, and microbiota in rats: implications for irritable bowel syndrome and psychiatric illnesses	Biological Psychiatry
8	85	Clarke G	2013	The microbiome-gut-brain axis during early life regulates the hippocampal serotonergic system in a sex-dependent manner	Molecular Psychiatry
9	83	Neufeld KM	2011	Reduced anxiety-like behavior and central neurochemical change in germ-free mice	Neurogastroenterology and Motility
10	83	Jiang HY	2015	Altered fecal microbiota composition in patients with major depressive disorder	Brain, Behavior, and Immunity

**Figure 6:**
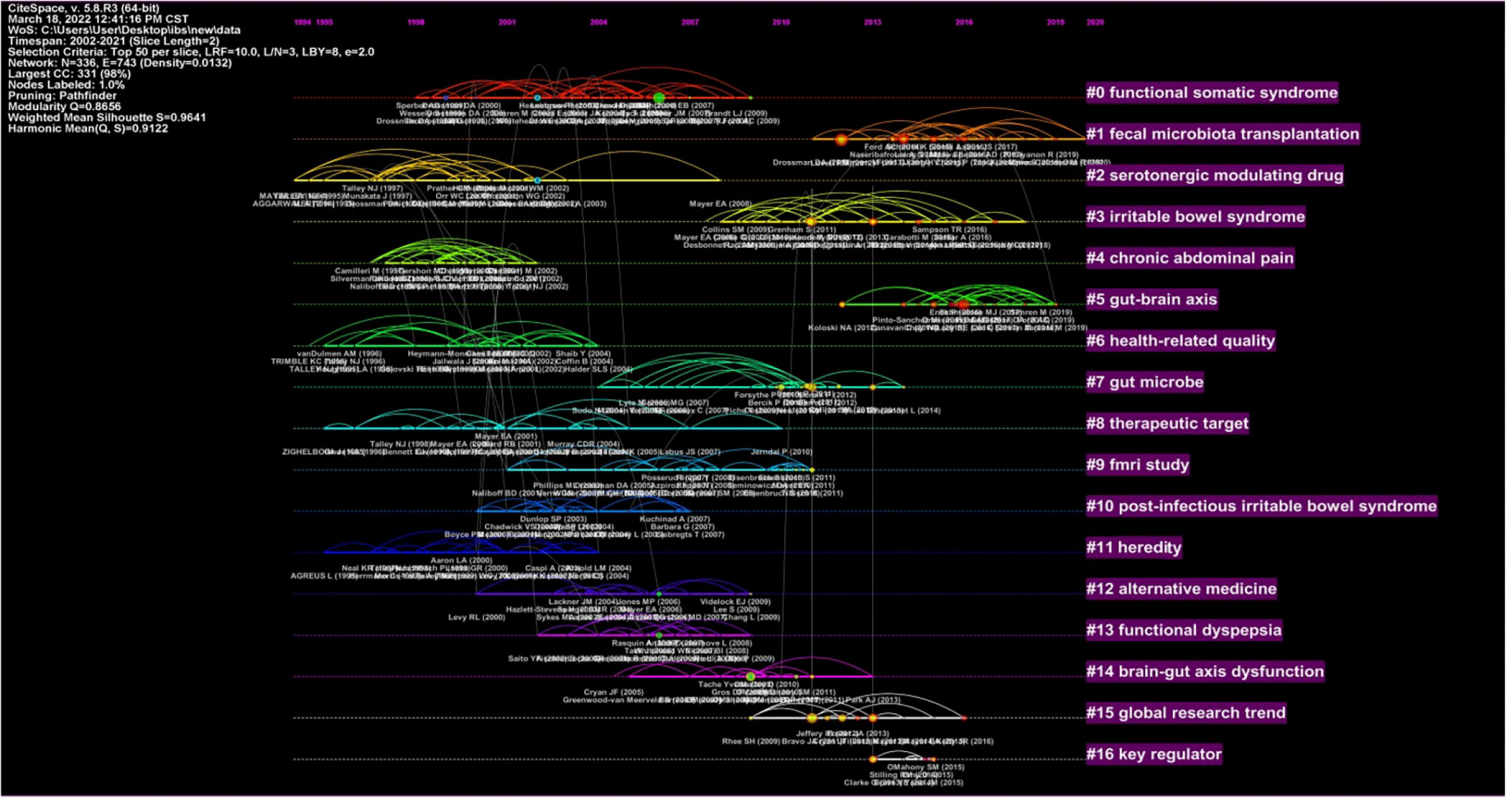
Timeline view for references co-citation clusters.

In this section, a network of 336 co-cited references was also generated by sampling the citation behavior of the top 50 articles per 2 years for the period between 2002 and 2021. Pathfinder was applied to prune the emerged network before the network was finally synthesized. For a better understanding of the characteristics inherent in cited studies on specific areas, cluster analysis was performed and clusters were determined according to the citation instances made by the top 50 most cited articles per 2 years. The network of 336 references was divided into 17 clusters and labeled using terms extracted from the titles of the articles in these clusters. The modularity Q score equaled to 0.8656, and the weighted mean silhouette score equaled to 0.9641. To explicitly identify recent developments in researches related to IBS and anxiety/depression since 2002, timeline visualizations based on the combined dataset are displayed in Figure 6, with the labels of clusters located on the right of the figure. The largest cluster was cluster #0 *functional somatic syndrome*, followed by cluster #1 *fecal microbiota transplantation* and cluster #2 *serotonergic modulating drug*. Themes such as serotonergic-modulating drug, health-related quality, therapeutic target, and heredity were popular in the early studies. Mid-term studies included fMRI study, post-infectious irritable bowel syndrome, alternative medicine, and gut microbe. Clusters representing new developments since 2010 include #1 on fecal microbiota transplantation and #5 on gut–brain axis.

What should be noted was that the modularity Q score and the weighted mean silhouette score were two momentous indexes that could indicate the quality and validity of clustering. It was widely recognized that the outcome of the cluster analysis was convincing on the condition that the modularity Q score was greater than 0.5 and the weighted mean silhouette score was >0.7. In Figure 6, the modularity *Q* score is 0.8656 and the weighted mean silhouette score is 0.9641, which further verified the significance of the clustering structure and the reliability of this result.

### Analysis of keywords

The knowledge map of co-occurrence keyword can reflect popular topics, while the burstness of keywords is considered as a valuable indicator of cutting-edge topics. The keyword co-occurrence map resulted in 655 nodes and 4,663 links. The nodes in the map present keywords, and the size of each node corresponded to the co-occurrence frequency of the keyword. As shown in Table 6, “irritable bowel syndrome,” “depression,” “quality of life,” “anxiety,” “symptom,” “disorder,” “prevalence,” “functional gastrointestinal disorder,” and “stress” were the top keywords with the maximum frequency and the highest centrality. Except for keywords such as “irritable bowel syndrome,” “depression,” and “anxiety,” which were search terms in this study, other keywords ranked ahead such as “quality of life” actually reflect hotspots and topics that were in focus in the field.

**Table 6:** Top 10 keywords in terms of frequency and centrality in the research.

**Rank**	**Frequency**	**Keyword**	**Rank**	**Centrality**	**Keyword**
1	1,648	Irritable bowel syndrome	1	0.55	Irritable bowel syndrome
2	552	Depression	2	0.08	Depression
3	521	Quality of life	3	0.07	Anxiety
4	463	Anxiety	4	0.06	Quality of life
5	438	Symptom	5	0.05	Symptom
6	387	Prevalence	6	0.05	Disorder
7	318	Disorder	7	0.05	Functional gastrointestinal disorder
8	278	Functional gastrointestinal disorder	8	0.04	Prevalence
9	194	Stress	9	0.04	Stress
10	178	Gut microbiota	10	0.04	Double blind

**Figure 7:**
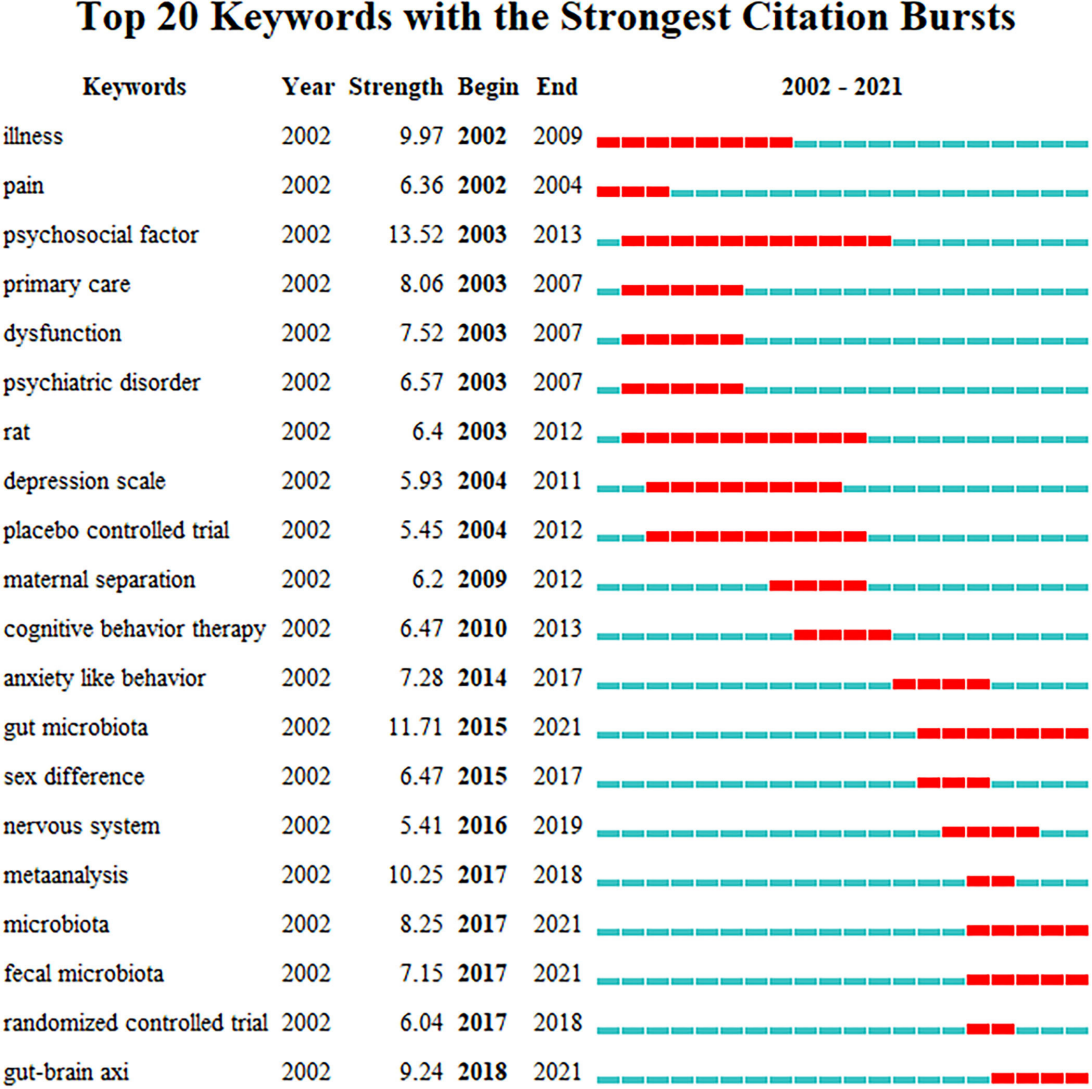
Top 20 keywords with strongest strength of burst.

Burst detection was applied to keywords in terms of the growth rate of their citations. The burstness of keywords is a valuable indicator of most active research topics and could help predict the research frontier. Figure 7 presents the top 20 keywords with the strongest strength of burst. The blue line represents the time interval, and the red line segment represents the period time in which a keyword was found to have a burst. For example, at the top of the list, the keyword “illness” had a period of burst between 2002 and 2009 with burst strength of 9.97. The keyword “psychosocial factor” had the longest period of 10 years and the largest burst strength of 13.52. The keyword “gut microbiota,” with the second strongest citation burst (burst strength of 11.71), began its burst in 2015. Moreover, keywords starting their bursts since the latest 5 years include “meta-analysis” (burst strength of 10.25), “microbiota” (burst strength of 8.25), “fecal microbiota” (burst strength of 7.15), “randomized controlled trial” (burst strength of 6.04), and “gut–brain axis” (burst strength of 9.24).

## Discussion

### General information

This study was conducted using data collected from WoSCC over the last 20 years to reveal the associated research focuses and frontiers. All told there were 2,562 publications related to IBS with anxiety or depression, and the growth trend showed a smooth rise in the number of publications with maximum publications in 2021 (227). As demonstrated in Figure 1, the number of documents published in 2002–2011 added up to 769, accounting for 30% of the total. However, four out of 10 articles (40%) were published over the last 5 years (2017–2021). Research related to IBS and anxiety/depression had started to become more topical, evidenced by an increasing number of articles.

A total of 2,562 publications retrieved in this study came from 66 countries. Among these countries, 12 contributed with articles more than 100 over the past two decades. The USA was the most productive country with a total of 833 publications, followed by China, the UK, and Sweden. It is evident that developed countries, led by the USA, had made decent contributions and play a more prominent part in the research areas associated with IBS and anxiety or depression. Furthermore, China, standing only behind the USA in the rank of publications number, is the sole developing country among the top 10 contributing countries and is also the main research power in this field. The analysis of the international research collaborations revealed a broad network of global collaborations (Figures 2, 3). There is reason to believe that close collaborations between countries and institutions had contributed to the continuous progress in this field. The most prolific author was Cryan JF from Ireland whose team devoted to conducting research in the field of psychobiotics, while Drossman DA was the top cited author, primarily due to his considerable insight into the relationship between IBS and gut–brain interaction, and the promotion of the further understanding of the functional gastrointestinal disorder.

### Research focuses on IBS research related to anxiety or depression

The keyword “gut–brain axis” is presented in Figure 7 with the latest citation bursts, and the keyword “gut microbiota” is presented with the highest citation bursts in the last decade. The high comorbidity of psychological disorders in gastrointestinal disorders suggests a close and complex connection between the brain and the gut. Previous studies have found that there is a two-way communication between the central nervous system (CNS) and the enteric nervous system (ENS), and the connection is named as the gut–brain axis. As the interest in gut microbiota increased, more and more studies have found that gut microbes are a key link in brain–gut interaction. Dysfunction in the microbiota–brain–gut axis is considered to be related to the occurrence and development of IBS. Gut microbiota, IBS symptoms, and psychology are interrelated and mutually influencing. Liu et al. (25) had discovered similar changes in fecal microbiota in diarrhea-predominant IBS (IBS-D) and depressive patients. Changes in gut microbes and their metabolite could probably account for the high comorbidity of psychological disorders in IBS patients. A recent systematic review of 17 studies reported a lower diversity among individuals with IBS and high anxiety or depression symptoms compared to controls and IBS-only cohorts. Meanwhile, IBS patients with anxiety or depression were more likely to have higher abundance of Proteobacteria, Bacteroides, Prevotella/Prevotellaceae, and lower Lachnospiraceae, according to the same study (26). Palma and his working team transplanted fecal microbiota from IBS patients with diarrhea to mice and observed symptoms of IBS and anxiety in mice who received fecal microbiota transplantation later, which also evidenced that the gut microbiota can not only alter pain modulation processes, but also interfere with host psychology, emotion, and behavior (27). Although the microbiota–gut–brain axis was considered to be involved in the regulation of digestion, modulation of immune system functions, and coordination of physical and emotional states among IBS patients mainly *via* endocrine, neural, and metabolic pathways, the mechanisms of IBS associated with anxiety or depression induced by microbiota–gut–brain axis dysfunction still need further study.

In Figure 6, it is obvious that cluster #1 *fecal microbiota transplantation* contained most of the documents published in recent years. Fecal microbiota transplantation (FMT) refers to a method of transplanting gut microbiota from one person into another. It was first documented about 1,700 years ago in China as a treatment for severe diarrhea and was well known all over the world for its excellent efficacy achieved in patients with refractory Clostridium difficile infection (CDI). Previous studies have demonstrated that the transplantation of microbiota could impact on various behavioral phenotypes such as anxiety-like and depression-like behaviors (28–30). With further research on IBS and the development of the theory of microbiota–gut–brain axis, the intestinal microbiota has been suggested as the pathophysiological basis of functional gastrointestinal disorders and psychiatric disorders, and FMT has been considered as an acceptable treatment option for IBS associated with anxiety or depression (31, 32). Studies had found that alterations in gut microbiota composition existed in patients with either IBS, depression, or anxiety, alone or comorbid (33), and FMT could not only improve gastrointestinal symptoms but also show meaningful benefits in relieving anxiety and depression. Most scholars believed that the multiple effects of FMT on IBS patients with anxiety or depression should be attributed to the changes in the diversity and composition of the gut microbiota (34–36). Studies reported that the relative abundance of intestinal flora was higher after FMT (36), beneficial bacteria such as Bacteroidetes, Firmicutes, Verrucomincrobia, and Euryarchaeota were improved (36, 37), and abundance of Faecalibacterium, Eubacterium, Escherichia, Enterobacteriaceae, Bacteroides, and Escherichia–Shigella was decreased (35, 36). Moreover, an open-label observational study reported psychiatric symptom change during FMT in adult patients with IBS. It showed that alpha diversity had increased after FMT in the patients with Hamilton Depression Scale (HAM-D) ≥8, and there was a correlation between the increase in the diversity of the microbes and the improvement of depression scores after FMT (38). Some researchers also believed that many patients with IBS maintained a response during their long-term follow-up after FMT, even though FMT effects decreased over time (39–41). However, there were differences in the outcomes of randomized controlled trials (RCTs) of FMT in IBS as some RCTs showed no effect on FMT treatment (42, 43). The contradictory results might be attributed to the factors including differences in the donor selection, differences in the patient selection, dose and frequency of FMT, and administration route (44, 45).

Alternative medicine is a branch of complementary and alternative medicine (CAM) which is subdivided into two categories, natural products and mind–body medicine. Dietary supplements, such as herbs and vitamins, are classified as the former. Mind–body medicine mainly includes hypnotherapy and cognitive behavioral therapy. It was reported that over one-third of IBS patients seek help from CAM for their bowel symptoms (46–48), and CAM was getting popular among IBS patients primarily because of its safety and perceived effectiveness (46, 47). More researchers focused on herbal medicine (49), and meta-analyses had indicated that herbal medicine was effective in relieving IBS symptoms, although the quality of evidence was low (50, 51). Lackner and Jaccard believed a constellation of CBT-specific and nonspecific processes that reciprocally interacted with each other in very complex ways to help patients achieve symptom relief and maintain remission (52). As for IBS patients suffering from anxiety or depression, mind–body intervention was believed to play a role *via* gut–brain axis, alleviating comorbid psychological distress implicated in the worsening of bowel symptoms and quality of life (53–55). Cognitive behavioral therapy (CBT) is a type of mind–body intervention which has the most empirical support. A randomized trail targeting 436 Rome III-diagnosed IBS patients revealed that CBT was more likely to improve patients' symptom and maintain gains long term, comparing with IBS education (56). However, one weakness of CBT is that it is time- and effort-consuming for therapists, and there is limited availability of competent therapists as well. Therefore, telephone-based and web-based CBT were carried out in recent years. Studies had illustrated that both telephone-based and web-based CBT had positive impacts on patients' understanding of IBS, symptoms, and quality of life (57, 58).

Since anxiety and depression are the most common psychological conditions in patients with IBS, classifying and subgrouping IBS patients based on both gastrointestinal symptoms and psychological profiles seem to be logical and it might be useful in directing treatment (59). Furthermore, early psychological evaluation and intervention should be considered in patients with IBS. Researchers should place more emphasis on scales, the most powerful tool to evaluate the mental health status, in clinical assessment and the regular screening for IBS patient. In addition, a growing number of studies have pointed out that antidepressants and some psychological therapies are effective treatments for IBS (18), so a multidimensional treatment approach for patients with IBS seems indispensable. At last, owing to the large amount of interindividual variability exists in symptom experiences of persons with IBS and the diverse causes of psychological disorders which are closely related to the initiation, development, and progression of IBS, personalized interventions could be of great benefit (60–62).

### Strength and limitation

Visualization of the knowledge map helps to highlight the key point of the research subjects, hotspots, and development trends in the field of IBS associated with anxiety and depression. We identified other bibliometric articles referring to specific psychology topics and others on functional gastrointestinal disorders, but no specific bibliometrics relating anxiety/depression to IBS; consequently, this article is a novel one in this topic. However, this study had a few limitations. Firstly, data were based on the Web of Science database solely, which is one of the most crucial and frequently used databases for bibliometric analyses. However, it might not contain all publications relevant to this study. Secondly, only publications in English were included in this study, which might cause the omission of some influential documents. Additionally, the quality of publications included might vary greatly, which may undermine the credibility of the results.

## Conclusions

A comparative bibliometric analysis was conducted *via* CiteSpace and GraphPad Prism in this study to provide information regarding research hotpots on IBS associated with depression or anxiety in the last two decades. The number of publications related to depression or anxiety research in the field of IBS has been steadily growing. The USA was the most productive country, and the University of California, Los Angeles, produced the most publications. Neurogastroenterology and Motility was the journal with the most publications in this field. Cryan JF and Drossman DA were the most influential authors. Gut–brain axis, fecal microbiota transplantation, and alternative medicine were the main current research. Although this study had certain limitations, it still provides a comprehensive insight into IBS associated with anxiety or depression. Also, it helps to provide information for researchers regarding research trends, topics, and potential collaboration with other authors, countries, and institutions.

## Data availability statement

The original contributions presented in the study are included in the article/supplementary material, further inquiries can be directed to the corresponding author.

## Author contributions

YC and ZH designed this study. YC performed the search and contributed to the manuscript writing. BL and PL collected and rechecked the data. SY performed the analysis. All authors reviewed and revised the manuscript.

## Funding

This work was supported by the Key Field Project of Ordinary Colleges and Universities in Guangdong Province (No. 2020ZDZX3011).

## Conflict of interest

The authors declare that the research was conducted in the absence of any commercial or financial relationships that could be construed as a potential conflict of interest.

## Publisher's note

All claims expressed in this article are solely those of the authors and do not necessarily represent those of their affiliated organizations, or those of the publisher, the editors and the reviewers. Any product that may be evaluated in this article, or claim that may be made by its manufacturer, is not guaranteed or endorsed by the publisher.
